# Recovery Responses of Central Hemodynamics in Basketball Athletes and Controls After the Bruce Test

**DOI:** 10.3389/fphys.2020.593277

**Published:** 2020-11-12

**Authors:** Yahui Zhang, Lin Qi, Frans van de Vosse, Chenglin Du, Yudong Yao, Jianhang Du, Guifu Wu, Lisheng Xu

**Affiliations:** ^1^College of Medicine and Biomedical Information Engineering, Northeastern University, Shenyang, China; ^2^Department of Cardiology, The Eighth Affiliated Hospital of Sun Yat-sen University, Shenzhen, China; ^3^Guangdong Innovative Engineering and Technology Research Center for Assisted Circulation, Shenzhen, China; ^4^Department of Biomedical Engineering, Eindhoven University of Technology, Eindhoven, Netherlands; ^5^Department of Physical Education, Northeastern University, Shenyang, China; ^6^Department of Electrical and Computer Engineering, Stevens Institute of Technology, Hoboken, NJ, United States

**Keywords:** central hemodynamic, acute response, 1-h recovery, bruce test, basketball athletes

## Abstract

**Purpose:**

It is commonly believed that central hemodynamics is closely associated with the presence of cardiovascular events. However, controversial data exist on the acute response of competitive sports on central hemodynamics. Moreover, the central hemodynamic response to exercise is too transient to be investigated. Therefore, this study aimed to investigate the central hemodynamic response in young basketball athletes and controls after 1 h recovery after exercise.

**Methods:**

Fifteen young basketball athletes and fifteen aged-matched controls were recruited to perform the Bruce test. Central hemodynamics were measured and calculated, including heart rate (HR), aortic systolic, diastolic, and pulse pressure (ASP, ADP, and APP), ejection duration (ED), sub-endocardial viability ratio (SEVR), central augmentation index (AIx), and AIx@HR75. Intra-group and inter-group differences were analyzed by two-way repeated measures ANOVA.

**Results:**

ASP significantly decreased at 10 min after exercise in athletes, while it markedly declined at 15 min after exercise in controls (*p* < 0.01). Additionally, only in the athlete group, ADP significantly decreased at 50 min and at 1 h after exercise. AIx was also significantly reduced at 1–2, 20, 30, and 40 min after exercise (all *p* < 0.05). Moreover, there were significant differences in the changes of these parameters between the two groups at these measurement points (*p* < 0.05). SEVR significantly recovered to the baseline level after 30 min, while ED and HR returned to baseline levels at 40 min after exercise in both groups.

**Conclusion:**

Sustained decrease of aortic BPs was sooner after the cessation of exercise in athletes than in controls, and changes of aortic stiffness were more evident in athletes than those in controls during the 1 h recovery period. Additionally, SEVR returned to the baseline sooner than ED and HR in athletes.

## Introduction

Central hemodynamics, including myocardial perfusion, wave reflection, and ascending aortic blood pressure, have been recognized as independent predictors of cardiovascular risk, which, in turn is closely associated with cardiovascular morbidity and mortality (Myers, 2003; Pini et al., 2008; Vlachopoulos et al., 2010a). Among them, myocardial perfusion, an indicator of supply and demand balance of the heart, reflects the coronary flow reserve (Chemla et al., 2009; Tsiachris et al., 2012); while wave reflection is an important marker of arterial stiffness (Laurent et al., 2011). Aortic blood pressure is more relevant than peripheral blood pressure to the pathogenesis of cardiovascular diseases (Chirinos et al., 2005; Agabitirosei et al., 2007).

It is well established that regular exercise training has a favorable effect on cardiovascular health and reduces the incidence and mortality of cardiovascular disease (Myers, 2003; Gielen et al., 2010). However, whether exercise training of different types has a favorable effect on central hemodynamics in athletes remains controversial. Endurance exercise can reduce the arterial stiffness index in ultra-endurance and endurance athletes (Knez et al., 2008). Additionally, differences in the effect of moderate exercise between endurance and resistance-trained athletes have been demonstrated by Hoonjan et al. (2013) who found that, in the endurance-trained group arterial stiffness (measured by carotid-femoral pulse wave velocity) increased after exercise and that this effect was not seen in the resistance-trained group (Hoonjan et al., 2013). By contrast, there were no effects of marathon exercise on wave reflection index and arterial stiffness in athletes (Vlachopoulos et al., 2010b). It has been reported that resistance training increased the central blood pressure and wave reflection index in strength-trained athletes (Otsuki et al., 2007). However, Heffernan et al. (2007) found that there was no effect of resistance exercise on arterial function and central blood pressure. Furthermore, a combination of endurance training and resistance exercise had a negative effect on the augmentation index (AIx) and central blood pressure (Hoonjan et al., 2013; Franzen et al., 2016).

Competitive basketball provides a special stimulus different from the above-mentioned forms of exercise. It is not the only type of exercise that combines endurance and resistance exercise training, but it also requires high levels of power, speed, and coordination. Some studies have reported that this special group of athletes has a higher incidence of sudden death, which is associated with abnormal hemodynamics (e.g., cardiomyopathy) (Harmon et al., 2015). However, few experimental studies have investigated the cardiovascular function of basketball athletes.

Acute exercise intervention can effectively highlight the response of cardiovascular function (Zhang et al., 2018). Liu et al. (2015) investigated the acute effects of cycling intervention on carotid arterial hemodynamics between basketball athletes and controls. The decreased stiffness of carotid artery in the basketball athletes was observed. Some studies have reported no effect on wave reflection index and arterial stiffness after acute marathon exercise (Vlachopoulos et al., 2010b). The central blood pressure and wave reflection index in strength-trained athletes were increased during resistance exercise (Otsuki et al., 2007).

However, the response of some cardiovascular parameters during exercise is too transient to be easily monitored and investigated (Pierce et al., 2018). Furthermore, the response of cardiovascular function immediately after exercise can be regarded as a predictor of mortality (Cole et al., 1999). Therefore, it is important to investigate changes in cardiovascular function during recovery. Furthermore, the effect of exercise training on central hemodynamic parameters during recovery is still controversial. It has been reported that central blood pressure (BP) and wave reflection significantly decreased after running a marathon (Vlachopoulos et al., 2010b). Carotid BP and augmentation index (AIx) markedly decreased at 30 min after low-resistance exercise, while there were no significant differences in pulse pressure (Okamoto et al., 2014). A study reported that exercise had no effect on AIx and aortic systolic pressure (ASP) after 10 min of recovery (Heffernan et al., 2007), while a higher AIx was observed during 15 min recovery in rowers (Franzen et al., 2016).

Based on the above problems, we aimed to investigate and compare the central hemodynamic response between young basketball athletes and age-matched controls at rest and during 1 h recovery after exercise.

## Materials and Methods

### Participants

Fifteen young basketball athletes and fifteen age-matched university students between 19 and 21 years old were recruited into this study. The young basketball athletes were from a professional basketball team belonging to the Chinese University Basketball Association (CUBA), which is the highest-level college league in China, providing reserve talents for the China Basketball Association (CBA). These basketball players were selected to participate in CUBA through national high-level testing. The frequency of competition is 7–8 games per year, including CUBA and other basketball tournaments. These athletes received regular training of 15.1 ± 3.5 h/week for 7 ± 2 years. The training activities involved endurance, strength, speed, skill, and tactics. Among them, the strength training intensity was 7–8 h/week, which mainly included an exhausting strength training and 3–4 sessions of equipment training. The controls were students from the College of Medicine and Biological Information Engineering of the Northeastern University, China. They did not engage in any regular exercise program. All participants had no history of cardiovascular disease and were normotensive (BP < 140/90 mmHg). These participants were fully informed of the study purpose and risks of the Bruce test before signing the written informed content. The investigation was approved by the Ethics Committee of the Northeastern University, China.

### Study Design

**FIGURE 1:**
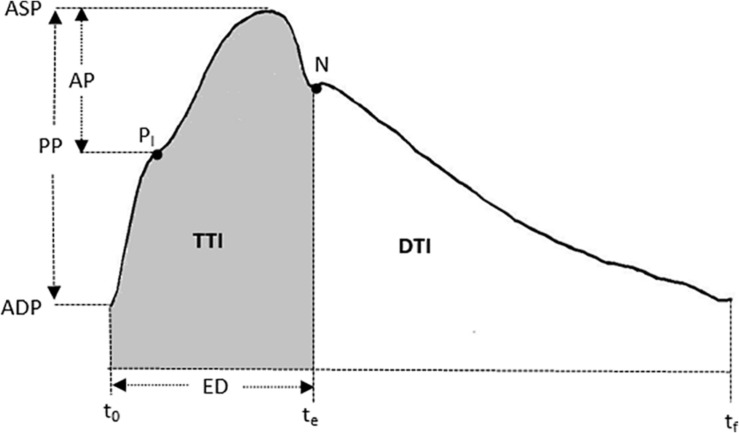
A typical central (ascending aortic) pressure waveform. The AIx = AP/PP. AP, augmentation pressure; PP, pulse pressure; PI is the pressure of inflection point. ASP, aortic systolic pressure; ADP, aortic diastolic pressure; ED, ejection duration. t_0_ is the time at the foot. t_e_ is the notch of aortic pressure waveform. t_f_ is the end time of an averaged waveform. *N* is the notch point. SEVR, DTI/TTI. DTI, diastolic time index; TTI, tension time index.

All measurements were conducted in the morning with each participant performing sessions at the same time of day (3 h after a meal) to minimize any potential diurnal variation. Before the experiment, all participants were required not to ingest any food or flavonoid-containing beverages and ethanol after midnight, and to avoid alcohol, caffeine, and exercise for at least 24 h prior to the measurements. Their hemodynamic data were collected in the Cardiovascular Function Laboratory. The baseline measurements were performed for both groups in the supine position after 10 min relaxation. Peripheral systolic blood pressure (SBP) and diastolic blood pressure (DBP) were measured using a Riva-Rocci sphygmomanometer, while the central hemodynamics, including heart rate (HR), ASP, aortic diastolic pressure (ADP), aortic pulse pressure (APP), ejection duration (ED), sub-endocardial viability ratio (SEVR), central AIx, and AIx@HR75, were obtained by measuring the radial artery waveform at the wrist, using applanation tonometry (SphygmoCor; AtCor Medical, Sydney, Australia) (Figure 1).

After acquiring baseline values of the central hemodynamic parameters, all participants underwent a treadmill test (EDAN TM-400, Shenzhen), using the standard Bruce’s treadmill protocol. This is one of the most common exercise protocols used in routine clinical tests and requires a high level of energy expenditure (Ellestad et al., 1969). The Bruce test is a maximal exercise test where the athlete works to complete exhaustion as the treadmill speed and incline is increased every 3 min. The procedures of testing are strictly in accordance with the statement from the American Heart Association (Fletcher et al., 1995; Franklin et al., 2001).

**FIGURE 2:**
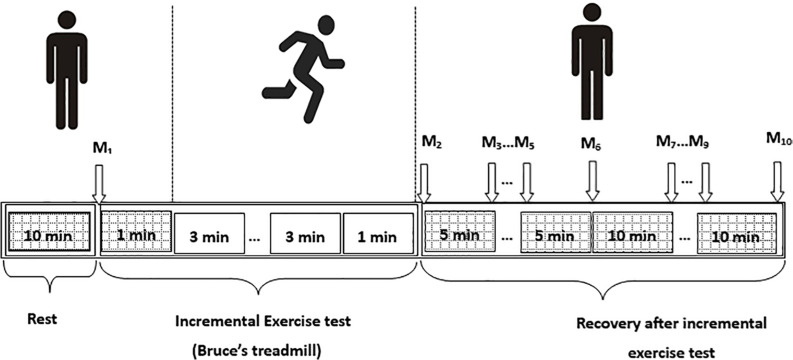
Schematic presentation of the experimental protocol. M_1_–M_10_ are the measurements before exercise and at 1–2, 5, 10, 15, 20, 30, 40, 50, and 60 min after exercise, respectively.

Following the Bruce test, all participants were asked to lie down on the measurement couch again and the peripheral SBP and DBP were measured. Basic subject information and these BP values were recorded in the SphygmoCor system. The radial artery waveform was then measured at the wrist using the applanation tonometry mode of the SphygmoCor system to obtain the abovementioned central hemodynamic variables. These were acquired at 1–2, 5, 10, 15, 20, 30, 40, 50, 60 min after exercise. In total, there were 10 measurements for each individual participant. It took about 1–2 min to complete one acceptable measurement with analyzable data. A schematic representation of the experimental protocol is shown in Figure 2.

### Statistical Analysis

All data are reported as Mean ± *SD*. Normal distribution for all the central hemodynamic variables was assessed by the Kolmogorov-Smirnov test. The independent *t*-test was used to compare differences in the basic characteristics of the two groups. Intra-group differences (before exercise and at 1–2, 5, 10, 15, 20, 30, 40, 50, 60 min after exercise) were analyzed by repeated measures ANOVA, and *post-hoc* analysis was used to determine differences between the different periods. Between-group differences of hemodynamic variables before and in 1 h recovery after exercise were analyzed by two-factor ANOVA. Intraclass correlation coefficients (ICC) were used to evaluate the reliability of measuring for central hemodynamic parameters in this study, and the ICC of all parameters were above 0.9. SPSS version 20.0 (IBM SPSS Statistics, United States) was used for all statistical tests, and *p* < 0.05 was taken as the criterion of statistical significance.

## Results

### Participant Information Between the Basketball Athletes and Controls

The basic participant information of the young basketball athletes and the controls are shown in Table 1. There were no significant differences in age, height, weight, and BMI between the two groups (all *p* > 0.05). Three basketball athletes and three controls were excluded for future data analysis due to incomplete tests or poor data quality.

**TABLE 1:** Information of young basketball athletes and the controls.

	**Athletes (*N* = 12)**	**Controls (*N* = 12)**	***p*-value**
Age (years old)	19.4 ± 1	19.8 ± 1	0.88
Height (cm)	189 ± 7	188 ± 2	0.44
Weight (kg)	86.2 ± 11.7	84.1 ± 17.0	0.73
BMI (kg/m^2^)	23.9 ± 1.9	23.8 ± 4.7	1.0

*Values are mean ± SD; Significance p-value < 0.05. BMI, body mass index.*

### Response of Central Blood Pressure and Heart Rate During Recovery

**FIGURE 3:**
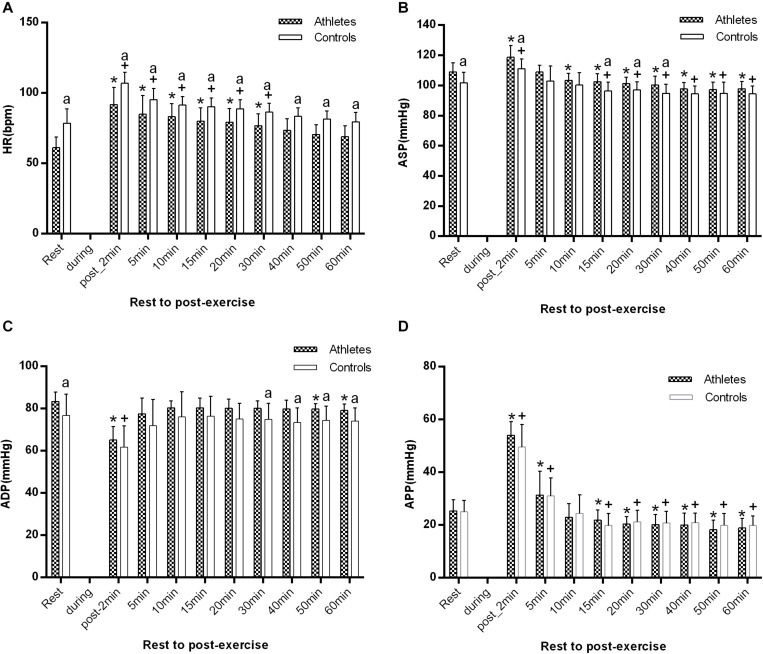
The difference of HR **(A)**, ASP **(B)**, ADP **(C)**, and APP **(D)** between athletes and controls at pre-exercise and 1–2, 5, 10, 15, 20, 30, 40, 50, and 60 min after exercise. The error bars represent the between-participant standard deviation (*SD*). The “^a^” indicates significant difference between the two groups with *p* < 0.05; The “*” and “^+^” indicate significant difference between pre- and post-exercise with *p* < 0.05 in athletes and controls, respectively.

As shown in Figure 3, baseline HR before the start of the exercise was significantly lower in athletes than in controls (61.1 ± 7.7 vs. 78.8 ± 10.2 bpm, *p* = 0.000), while ASP and ADP were significantly higher in athletes (109 ± 6.1 vs. 101.8 ± 6.9 mmHg, *p* = 0.01; and 83.4 ± 4.4 vs. 76.8 ± 10.1 mmHg, *p* = 0.048). However, there was no significant difference in APP between the two groups (*p* > 0.05). Immediately after exercise (1–2 min post-exercise), in both groups, HR, ASP, and APP were significantly higher in comparison with the baseline level, while the ADP was significantly lower (all *p* < 0.05).

During the 1 h recovery period, HR was significantly lower in athletes than in controls at all measurement times (all *p* < 0.05, Figure 3A), and at 40 min after exercising it had returned to the baseline level in the two groups. ASP significantly decreased at 10 min after exercise in the athletes, while it markedly declined at 15 min after exercise in controls (*p* < 0.01). There was a significant difference from 15 to 30 min after exercise between the two groups (all *p* < 0.05, Figure 3B). ADP was only significantly decreased, just in athletes, at 50 min, and it was significantly higher in athletes than in controls after 30 min of recovery (all *p* < 0.05 at 30, 40, 50, and 60 min post-exercise, Figure 3C). APP significantly decreased just after 15 min in both groups.

### Response of Cardiac Function During Recovery

**FIGURE 4:**
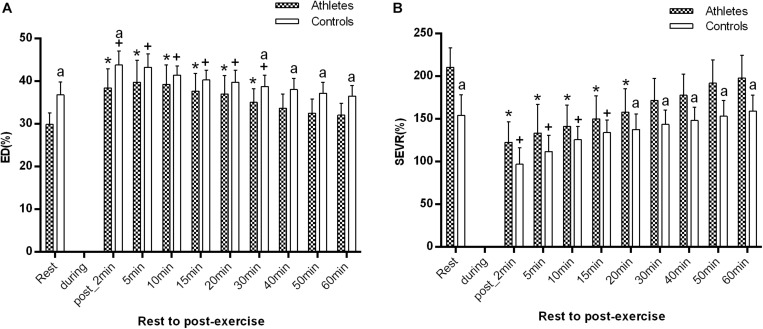
The difference of ED **(A)** and SEVR **(B)** between the athletes and controls before and after exercise. The error bars represent the between-participant standard deviation (*SD*). The “^a^” indicates significant difference between the two groups with *p* < 0.05; The “*” and “^+^” indicate significant difference between pre- and post-exercise with *p* < 0.05 in athletes and controls, respectively.

As shown in Figure 4, the baseline ED in basketball athletes was significantly lower (29.9 ± 2.7% vs. 36.8 ± 3.0%, *p* = 0.000), while SEVR was significantly higher (210.5 ± 22.8% vs. 154.2 ± 24.0%, *p* = 0.000) in comparison with controls.

ED significantly increased at 1–2 min after exercise, while SEVR significantly decreased in both groups (all *p* < 0.05, Figure 4A). ED reached the maximum value at 5 min post-exercise in the young basketball athletes, but at 1–2 min post-exercise in controls. SEVR was significantly higher in athletes than in controls, while ED were significantly lower (all *p* < 0.05, Figure 4).

During recovery, ED was significantly lower in athletes at 30, 40, 50 min, and 1 h after exercise, while SEVR was significantly higher in athletes at 20, 30, 40, 50 min, and 1 h after exercise than that in controls (both *p* < 0.05 from 30 min to 1 h post-exercise, respectively). In addition, in both groups, ED and SEVR gradually recovered to the baseline level at 40 and 30 min, respectively.

### Response of Aortic Stiffness During Recovery

**FIGURE 5:**
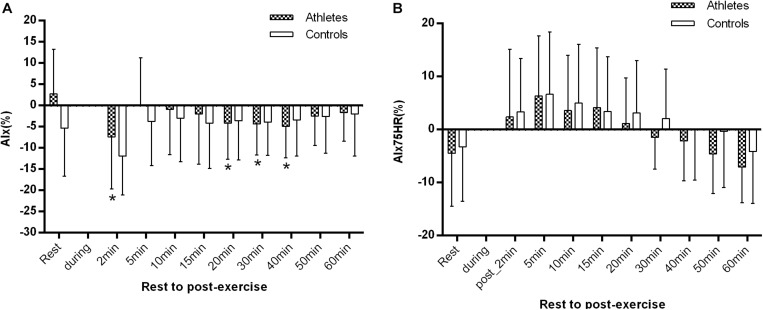
The difference of AIx **(A)** and AIx@HR75 **(B)** between athletes and controls before and after exercise. The error bars represent the between-participant standard deviation (*SD*). The “^a^” indicates significant difference between the two groups with *p* < 0.05; The “*” and “+” indicate significant difference between pre- and post-exercise with *p* < 0.05 in athletes and controls, respectively.

There were no significant differences in AIx and AIx@HR75 in both groups at rest and at the other time points (all *p* > 0.05). However, AIx significantly decreased in athletes 1–2 min after exercise in comparison with the at rest value (−7.4 ± 12.2 vs. 2.8 ± 10.5%, *p* = 0.003, Figure 5A). In comparison with the baseline level, AIx was significantly reduced in athletes at 20, 30, and 40 min after exercise (all *p* < 0.05, Figure 5A). However, there was no significant difference in AIx@HR75 during the whole recovery period in controls (all *p* > 0.05, Figure 5B).

## Discussion

Our study has demonstrated that, in comparison with the controls, the young basketball athletes had lower HR and smaller ED, while they had higher ASP, ADP, and SEVR at rest. During the 1 h recovery period, ASP significantly decreased at 10 min after exercise in athletes, while it markedly declined at 15 min after exercise in controls. Additionally, ADP significantly decreased at 50 min and 1 h after exercise, only in the athlete group, and AIx was also significantly reduced at 20, 30, and 40 min after exercise. Meanwhile, SEVR was significantly recovered to the baseline level at 30 min, while ED and HR had returned to the baseline level at 40 min after exercise compared with the rest values in both groups.

It has been reported that aortic BPs are more relevant than peripheral BP to the pathogenesis of cardiovascular diseases (Chirinos et al., 2005; Agabitirosei et al., 2007; Huang et al., 2011). This study found that ASP was significantly higher in basketball athletes than that in controls at rest. Our previous study reported that basketball athletes had higher central BP than that in controls (Zhang et al., 2017), while Liu et al. (2015) found that basketball athletes had lower carotid BPs in comparison with controls. Some studies have reported that higher central BPs were observed in endurance athletes (Vlachopoulos et al., 2010b; Laurent et al., 2011) and the effects of combined exercise on central BPs also had been investigated (Liu et al., 2015; Franzen et al., 2016). Higher central BPs which have a negative effect on the cardiovascular system were considered to be associated with stroke volume, total peripheral resistance, ventricle ejection, and wave reflection (Laurent et al., 2011).

Although in the athletes, higher central BP was seen at rest, its sustained decrease occurred earlier than that in the controls. ASP significantly decreased at 10 min after exercise in athletes, while it markedly declined at 15 min after exercise in controls. More importantly, only in the athlete group was ADP significantly decreased and this did not occur until 50 min and 1 h after exercise. Some studies have found a sustained reduction in BP after exercise, i.e., post-exercise hypotension (Kenney and Seals, 1993). There have been few reports of central BPs after exercise in basketball athletes. Although a recent study has reported that carotid BPs tended to increase during a cycling intervention in basketball athletes, and were significantly higher than those in controls (Liu et al., 2015). On the other hand, it has been reported that aortic BP significantly decreased after running a marathon (Vlachopoulos et al., 2010b). Others have found that carotid BP was decreased at 30 min after low-resistance exercise, while there were no significant differences in pulse pressure (Okamoto et al., 2014). A study reported that exercise had no effect on ASP after 10 min of recovery (Heffernan et al., 2007). In addition, some have reported a rise in BP sustained for 12 h after a bout of exercise in patients with hypertension (MacDonald, 2002). Cardiac output and peripheral resistance which are influenced by cutaneous vasodilation led by thermoregulation, blood volume, sympathetic nerve activity, and nitric oxide are the main explanations of the potential mechanism of post-exercise hypotension (Hagberg et al., 1987; Franklin et al., 1993; Kulics et al., 1999; MacDonald, 2002).

AIx is a complex cardiovascular parameter, which is influenced by many central and peripheral factors, such as central and peripheral BPs, arteriolar vasomotion, ejection functions, VO_2_ peak and cf-PWV, and wave reflection (Nichols et al., 1991; Denham et al., 2016). AIx, an indirect indicator for assessing aortic stiffness, was also significantly reduced at 1–2, 20, 30, and 40 min after exercise in athletes, indicating that arterial stiffness may be improved after post-exercise vasodilation. Our previous study reported higher AIx values in athletes at rest (Zhang et al., 2017). However, there have been few studies investigating the acute effect on AIx in basketball athletes during and after exercise. Liu et al. (2015) recently reported the effect of cycling exercises on the carotid stiffness, and found that both the pressure-strain elastic modulus and arterial stiffness were significantly lower in basketball athletes.

These findings are not consistent with the results in this study. So we tried to find some evidence of the influence of other sports on the AIx, but the reported results are also contradictory (Wang et al., 2015). Some studies have reported that AIx was significantly lower in endurance athletes (Edwards and Lang, 2005; Denham et al., 2016), while others found that there was no significant difference in AIx between ultra-endurance marathon runners and controls (Knez et al., 2008; Gielen et al., 2010), or that AIx was significantly higher in rowers than in controls (Franzen et al., 2016). These observations may be also related to the effect of long-term excessive training on the changes of arterial and cardiac function (Liu et al., 2015). It has been reported that wave reflection was significantly decreased after running a marathon (Vlachopoulos et al., 2010b). AIx decreased at 30 min after low-resistance exercise, while there was no significant difference in pulse pressure (Okamoto et al., 2014). A study reported that exercise had no effect on AIx and ASP after 10 min of recovery (Heffernan et al., 2007), while a higher AIx was observed at 15 min after exercise in rowers (Franzen et al., 2016). Others have found that cf-PWV was associated with the corresponding changes in post-exercise attenuation of APP, and that this was influenced by attenuated wave reflection (Akazawa et al., 2015; Sugawara et al., 2015). In this study, APP was significantly increased immediately after exercise and then markedly decreased, especially in athletes. APP may be an important parameter related to AIx.

In this study, although the young basketball athletes had higher AIx, they had greater cardiopulmonary fitness (lower HR and ED), and stronger myocardial perfusion (higher SEVR) than those of the controls. HR, ED, and SEVR are also clinically important, reflecting cardiac blood supply and myocardial perfusion (Knez et al., 2008). It has been reported that lower baseline HR in athletes is associated with improved autonomic nervous system activities and cardiopulmonary fitness after long-term training exercise (Alentejano et al., 2010). It was also related to the longer cardiac relaxation time (diastolic time) and shorter ED (systolic time). Furthermore, the lower HR and shorter ED may contribute to a better balance between myocardial oxygen supply and demand, resulting in a better match in the coupling between the left ventricle and the vascular system (Duncker and Bache, 2008). SEVR was improved in athletes due to a better balance between myocardial oxygen supply and demand.

More importantly, in terms of the central hemodynamic response during 1 h recovery, SEVR significantly recovered to the baseline level after 30 min, while ED and HR were returned to baseline level at 40 min after exercise. It was observed that there were no significant differences in ED and SEVR between the two groups between 5 and 20 min, while there were significant differences from 20 min to 1 h after exercise. Some studies have reported that the recovery of cardiac function after exercise is mainly associated with athletes’ physical fitness and exercise intensity (Withers, 1977; Alentejano et al., 2010). It is likely that this recovery of cardiac function after exercise is associated with lipid peroxidation and antioxidant potential (Wonders et al., 2007). In addition, some studies have found that the decreased BP after exercise could be associated with changes in HR, and that the potential mechanisms are primarily related to changes in sympathetic nerve activity, cardiac filling, the production of nitric oxide, and myocardial contractility during recovery (Dujić et al., 2006; Saldanha et al., 2016). It is therefore important to have a more comprehensive assessment with a longer recovery time to better understand the decreased BP and AIx after exercise and HR, ED, and SEVR after 1 h of recovery.

Some potential limitations of the present study should be emphasized. Firstly, the participants were all young adults between 19 and 21 years old so that these findings cannot be generalized to older (or younger) individuals. Secondly, the number of participants was relatively small. Thirdly, biochemical parameters, such as plasma lipids, which may provide some underlying mechanisms for the observed functional changes during recovery from exercise, have not been measured in the present study.

## Conclusion

This study has demonstrated the differences of central hemodynamic responses (e.g., central blood pressure, central AIx, and myocardial perfusion) between young basketball athletes and controls during a 1 h recovery period following exercise. A sustained decrease of aortic BPs was sooner after the cessation of exercise in athletes than that in controls, and changes of aortic stiffness were more obvious in athletes than those in controls during the whole recovery period. Additionally, SEVR returned to baseline earlier than ED and HR in athletes. Although the athletes had higher central BPs and AIx, indicating high cardiovascular risks in comparison with controls at rest, faster recovery was seen during the 1 h recovery. The response and recovery time of central hemodynamic variables after exercise in athletes may be related to the higher cardiopulmonary fitness and stronger myocardial perfusion than those of the controls at baseline. Moreover, it may also be attributed to the regulation of stroke volume, total peripheral resistance, wave reflection, and pulse pressure during and after exercise.

## Data Availability Statement

The raw data supporting the conclusions of this article will be made available by the authors, without undue reservation, to any qualified researcher.

## Ethics Statement

The studies involving human participants were reviewed and approved by the Ethics Committee of the Northeastern University, China. The patients/participants provided their written informed consent to participate in this study.

## Author Contributions

YZ and LX proposed the scientific problems. YZ, LQ, and LX designed the experiments. YZ and CD collected the experimental data. YZ processed and calculated the data. YZ conducted statistical analysis and wrote the draft manuscript. JD, LX, FV, YY, and GW contributed to the revision and final version of manuscript. All authors contributed to the article and approved the submitted version.

## Conflict of Interest

The authors declare that the research was conducted in the absence of any commercial or financial relationships that could be construed as a potential conflict of interest.
